# An Africa point of view on quality and safety in imaging

**DOI:** 10.1186/s13244-022-01203-w

**Published:** 2022-03-26

**Authors:** Michael G. Kawooya, Harriet Nalubega Kisembo, Denis Remedios, Richard Malumba, Maria del Rosario Perez, Taofeeq Ige, Francis Hasford, Joanna Kasznia Brown, Miriam Mikhail Lette, Boudjema Mansouri, Dina H. Salama, Fozy Peer, Rose Nyabanda

**Affiliations:** 1 Ernest Cook Ultrasound Research and Education Institute, Kampala, Uganda; 2 Department of Radiology, Mulago National Referral Hospital, Kampala, Uganda; 3 Department of Clinical Radiology, Northwick Park Hospital, Harrow, HA1 3UJ UK; 4 Division of Healthier Population (HEP), World Health Organization, Geneva, Switzerland; 5 Department of Medical Physics, National Hospital, Abuja, Nigeria; 6 Medical Physics Department, School of Nuclear and Allied Sciences, University of Ghana, Accra, Ghana; 7 University of Bristol, Musgrove Park Hospital, Park Lane, Taunton, UK; 8 International Atomic Energy Agency, Vienna, Australia; 9 Faculty of Medicine of Algiers (Algeria), University Hospital of Bab El Oued, Bab El Oued, Algeria; 10 Radiology and Medical Imaging Technology Department, Misr University for Science and Technology, Giza, Egypt; 11 Society of Radiographers of South Africa, Pinelands, South Africa; 12 Diagnostic Services and Health Information, Kenyatta National Hospital, Nairobi, Kenya

**Keywords:** Radiology, Medical imaging, Quality and safety, Medical imaging education, Radiation safety

## Abstract

Africa has seen an upsurge in diagnostic imaging utilization, with benefits of efficient and accurate diagnosis, but these could easily be offset by undesirable effects attributed to unjustified, unoptimized imaging and poor quality examinations. This paper aims to present Africa’s position regarding quality and safety in imaging, give reasons for the rising interest in quality and safety, define quality and safety from an African context, list drivers for quality and safety in Africa, discuss the impact of COVID-19 on quality and safety, and review Africa’s progress using the Bonn Call for Action framework while proposing a way forward for imaging quality and safety in Africa. In spite of a healthcare setting characterized by meagre financial, human and technology resources, a rapidly widening disease-burden spectrum, growing proportion of non-communicable diseases and resurgence of tropical and global infections, Africa has over the last ten years made significant strides in quality and safety for imaging. These include raising radiation-safety awareness, interest and application of evidence-based radiation safety recommendations and guidance tools, establishing facility and national diagnostic reference levels (DRLs) and strengthening end-user education and training. Major challenges are: limited human resource, low prioritization of imaging in relation to other health services, low level of integration of imaging into the entire health service delivery, insufficient awareness for radiation safety awareness, a radiation safety culture which is emerging, insufficient facilities and opportunities for education and training. Solutions to these challenges should target the entire hierarchy of health service delivery from prioritization, policy, planning, processes to procedures.

## Key points

1. Global health themes increased interest for quality and safe imaging in Africa.
2. Collaboration, training and research are catalysts to quality and safety transition in Africa.
3. COVID-19 has affected quality and safety for imaging in Africa.
4. Technologies like machine learning and AI are key to enhance quality and safety.

## Introduction

This section explains the rising interest in quality and safety for imaging for Africa gives a contextual definition of quality and safety in imaging for Africa and lists Africa’s the drivers for quality and safety.

### Africa’s interest in imaging quality and safety

There has been a rising interest in quality and safety for imaging in Africa over the last ten years. This interest may be attributed to participation in global health themes and programs like sustainable development goals (SDGS), basic safety standards (BSSs), Bonn Call for Action (BCA), universal health coverage and the launch of Africa’s radiation safety campaign [1–5]. This is further compounded by a shift in disease burden exemplified by change in trends of neglected tropical diseases, maternal and childhood diseases, non-communicable diseases, trauma, cancer, HIV, tuberculosis and of recent the COVID-19 pandemic [6–8]. The launch of Africa’s radiation safety campaign, AFROSAFE.in 2015, enhanced awareness, mobilized and focused Africa toward imaging quality and safety [9]. In addition, forging of partnerships and collaboration with professional societies, global agencies and organizations like WHO and IAEA have also promoted interest in quality and safety.

## Definition of quality and safety from a radiological perspective

It is important to have an accurate definition of quality, which is contextualized to Africa, and to define the drivers of quality and safety in imaging for Africa. It is this contextualized definition that dictates the types of strategies to effectively improve quality and safety in Africa. There have been several definitions for quality, and some of the most popular definitions are comprised of effectiveness, safety, patient centeredness, timeliness, efficiency and equitability and integration. Busse et al. further classified the first three of these elements into health service and the last three as health-system determinants of quality and safety [10].

Of these six elements, effectiveness, safety, patient centeredness stand out as core elements [11]. Effectiveness is taken to refer to evidence-based practice, which in radiology is implemented through evidence-based standard guidelines and tools. Safety refers to the reduction of risks, errors and harms in health care, so as to tilt the balance towards benefits, which in radiology is majorly by application of the principles of justification and optimization through the use of clinical imaging guidelines (CIGs) and diagnostic reference levels (DRLs). Patient-centered health care refers to healthcare that is respectful and responsive to patient’s individual preferences, needs and values and ensuring that patient’s values guide all clinical decisions [12, 13]. This description of patient’s centered is also a component of evidence-based health care and is applicable to imaging since it is embedded within the justification principle. The recent emphasis on radiation benefit–risk dialogue with the patient at the center underscores patient-centeredness.

### The drivers for quality and safety within the African context

The six elements of quality and safety need to be contextualized to Africa, given Africa’s peculiar heathcare setting characterized by: large and predominantly young populations, low awareness and prioritization of imaging in healthcare, glaring health issues like shortages of drugs and vaccines. Africa’s other unique characteristics are: low- and-middle-income status, higher illiteracy levels, lower workforce numbers and imaging skills, rudimentary information technology infrastructure (ICT), lower digitization and automatization with older and at times outdated equipment, absent or unstable electricity-power sources. Africa is also burdened with less efficient health-administration and management systems, paucity of critical health data, rudimentary data acquisition, storage and management systems, and insufficient education and training opportunities and infrastructure [14]. Consequently, in a region where hardly any imaging services exist for rural populations with averages of 50–60% within most African countries, it is difficult to conceptualize and actualize elements of quality and safety like patient-centered care, timeliness, equitability and integration as one would for developed countries. Some form of imaging, accessible by the majority population, has first got to be in place before actualizing these concepts. Although Africa is one of the fastest urbanizing continents, she is challenged with unmanaged urbanization with poor urban infrastructure and its associated, negative per annum capita growth, weak investment and declining productivity [15]. With such stated results, unmanaged rural–urban migration may therefore not advantage populations with regard to access to quality and safe imaging.

Currently, patient-centeredness can be difficult to actualize in the context of absent, intermittent or very meagre imaging technologies and options. Patients cannot afford to choose between technologies, but take up whatever is available and affordable. Furthermore, the higher illiteracy levels in these rural and unmanaged urban areas make comprehension and informed decision-making often impossible; therefore, the approach is at times paternalistic with the patient taking the “the doctor knows what is best for me” position. The situation is at times further compounded by undiffused myths and misconceptions arising from cultural aspects and lack of information or illiteracy [16].

Equitability and integration in the context of absent, intermittent or very meagre imaging technologies and options is a challenge. Timeliness may be an enigma in a setting where most patients for several reasons report late for treatment with the disease in advanced stages and where lack of finances, transport and communication incapacitate travel. To cater for the scarcity in rural and unmanaged urban Africa, the immediate and initial steps in improving quality and safety is to make the service available, affordable and accessible. Busse adopted an approach to quality and safety improvement, which is based on the WHO 2006 Health Systems Framework, and comprises of the six building blocks, intermediate goals and overall goals where quality is defined as an intermediate goal [17]. We propose to modify this approach by adding immediate goals, before the intermediate goals. The proposed immediate goals are: availability, accessibility and affordability and safety. Once these are in place, we can then aim at intermediate goals like coverage. The final or overall goal is also contextualized to include efficiency, high-quality imaging, universal imaging-coverage, patient-centeredness, and financial risk protection. We adopt the WHO and Busse’s “building blocks’ which we term as “drivers” for the intermediate goal to also be the “building blocks” or drivers for the immediate goals [10, 17]. In addition, we modify these building blocks to contextualize to the African health care; these building blocks/drivers are:

- Planning and financing
- Administration-system efficiency
- Accountability for resources
- Auditable systems and services
- Adaptable systems and services
- Awareness creation
- Availing appropriate and affordable and technologies and accessories
- Automation of equipment and systems
- Amalgamating (integrating) imaging into the essential healthcare package.

These immediate goal drivers are also similar to the drivers for the transition from intermediate to overall goal but with variations in magnitude as need arises.

### The catalysts for quality and safety in the African setting

There are three main catalysts for speeding up the quality and safety transition, namely collaboration and partnerships with professional bodies, global agencies and organizations; education and training; research and innovation [18–20]. These three have played a major role in promoting quality and safety in Africa throughout the past decade and should be scaled up.

Table 1: Professional bodies/societies in selected African countries.

**Table 1:** Numbers of national professional societies in selected African countries

Professional societies in selected African countries					
Country	Radiologist	Radiographers	Sonographers	RO	Medical physicists
Kenya	1	1	1	0	0
Uganda	1	1	1	0	1
Tanzania	1	1	0	0	0
Nigeria	1	2	1	1	1
S. Africa	1	1	1	0	1
Ethiopia	1	1	0	0	0
Cameroon	1	1	0	1	0
Algeria	2	0	0	0	0
Ghana	1	1	1	0	1
Cote d'ivoire	1	1	0	0	0
Swaziland	0	0	0	0	0
Zimbabwe	1	2	1	0	0

RO-Radiation Oncologists

### IAEA partnerships as catalysts to quality and safety

The IAEA has contributed to the training and skilling of health workers for radiation safety through technical cooperation projects and workshops. The IAEA technical projects extend up to 45 countries in Africa [21]. Technical cooperation agreements are within the framework of the African Regional Co-operative Agreement for Research, Development and Training Related to Nuclear Science and Technology (AFRA), which came into force in April 1990 and was revised in April 2020.

The Lancet Oncology Commission on Medical Imaging and Nuclear Medicine whose role is to assess and statistically model the needs for imaging and nuclear medicine resources worldwide was convened by the IAEA in 2018 [22]. It launched the IMAGINE database, in 2019, listing available radiological resources for effective in-country planning of imaging and nuclear medicine technologies [23].

The IAEA also developed and promoted methodologies for comprehensive facility-based quality and safety audits for imaging and radiation therapy [7, 24–26].

### WHO partnerships as catalysts to quality and safety

Cognizant of the potential risk from inappropriate use of ionizing radiation in diagnostics and therapy in healthcare settings and the potential harm from this risk especially in children, WHO launched the Global Initiative on Radiation Safety in the Healthcare Settings in 2008 [27]. Within this initiative, and working with experts from Africa and other countries, a tool titled “Communicating Radiation Risks in Pediatric Imaging” was developed and following training, it is being applied in Africa [2]. Such a tool resulting from such a partnership is of importance to aid quality and safety for Pediatric Imaging. In addition, WHO rallied the International Society of Radiology and, radiology experts worldwide including Africa to come up with a “rapid” imaging guideline entitled “Use of Chest Imaging in COVID-19, A Rapid Advice Guide [28]. Such a call was meant to standardize the use of imaging for COVID-19 among partners including Africa for quality and safety.

In March 2021, the WHO updated and launched its guidelines on TB screening, scaling up the role of chest radiography and Computer-Assisted Detection (CAD) [29] globally, with Africa inclusive [30, 31]. Such updated guidelines are evidence-based to catalyze quality and safety among partners.

## Impact of COVID-19 on quality and safety for imaging in Africa

Unprecedented events like disasters and pandemics have a greater impact on health systems in developing countries more than the developed because of limited health resources and less developed infrastructure. African countries have far less coverage of imaging equipment compared to developing countries [32].

COVID-19 has had both positive and negative effects on quality and safety of imaging in Africa. A positive effect of COVID is underscoring the indispensability of imaging and the need to integrate into the essential healthcare package [33] in addition to the acceleration in application of E-learning technologies which favor training in radiation safety and quality [5].

Despite the positives noted above, there has been increased utilization of ionizing radiation for lung and chest imaging. Such increased utilization is not free of inappropriate application and related side effects resulting from limited human resource, patient overload and outdated equipment in most African health facilities [34]. In addition, given the limitations for Infection prevention and control measures coupled with lower vaccine coverage and uptake, patient-centeredness, timeliness, efficiency and equitability have been compromised especially in rural areas. Lockdowns and reduced incomes have also impacted patient travel to health facilities to seek health care services inclusive of imaging [35].

To foster quality and safety in COVID-related imaging, the WHO in partnerships with stakeholders including those from Africa developed the WHO-rapid imaging guideline [29] and kick started implementation [33]. This guideline was to ensure standardized practice for imaging procedures across the board.

Reviewing Africa’s progress on quality and safety within the framework of the Bonn Call for Action (BCA).

The BCA is a global goal focusing only on imaging and dealing largely with safety. This call lists different Actions related to safety and quality for utilization of ionizing radiation for diagnostic imaging. The next sections of this paper review Africa’s progress with the BCA [35, 36]. The next sections of this paper review Africa’s progress with the BCA.

Action 1: Enhancing implementation of justification of procedures.

This Action seeks to Introduce and apply awareness, appropriateness and audit, which are the tools that facilitate and enhance justification of imaging procedures.

Awareness has been identified as tool for highlighting the dangers and risk of inappropriate use of ionizing radiation. In Africa, studies have indicated that there is some awareness of such risk; according to an unpublished survey of awareness of special radiation risk for pediatric imaging in Africa showed that only 50% of pediatricians were aware of this special risk but there are indicators that awareness for radiation safety has improved over the past decade. Such awareness has been shown to improve with training and information provision most especially among referrers. This is evidenced by a study carried out in 2020 in a Uganda hospital to assess the impact of training of referrers on awareness in radiation protection showed that training of referrers could improve awareness by 30% [37].

The launch of AFROSAFE.rad in Nairobi in 2015 opened many doors for Africa to showcase its progress in quality and safety in imaging in regional and global scientific fora, becoming increasingly active and effective in regional and global campaigns, strategies and activities, with a multiplier effect on awareness.

One major highlight clearly showing increased awareness was when in 2015, the government of Kenya, Uganda, Malaysia and Spain sponsored the World Health Assembly (WHA) side event WHA68 with the theme “Imaging for Saving Kids; The inside Story about Patient Safety in Pediatric Radiology” [38]. Another evidence for progress in awareness is the increased research by scientists and students. Over the last ten years, several African undergraduate, postgraduate students and doctoral students have done research and published dissertations and theses on different aspects radiation safety [39, 40].

The third component under the first Action of the Bonn Call relates to implementing and regularly updating of clinical imaging referral guidelines (CIGs). Much development of CIG is a complex, expensive and vigorous activity; Africa has not been left behind in implementing CIGs [37]. There is evidence of the presence and application of the same as shown in unpublished data from a survey of 10 African countries showing that CIG was only available in 2 out of the 10 countries although all countries were willing to use CIGs. The initial efforts on introduction of CIG in Africa began in 2014. The International Radiology Quality Network (IRQN), now the ISR- International Society of Radiology Quality and Safety Alliance (ISRQSA) in partnership with WHO and world experts, Africa inclusive, in 2014 in released a set of 44 consensus guidelines entitled “Referral guidelines for diagnostic imaging”, which primed CIG usage in Africa. In partnership and support from the European Society of Radiology and the IAEA. Africa has now adopted the iGUIDE subsequent to pilot projects in Algeria, Cameroon, Cote D’Ivoire, Egypt, Kenya and Uganda [41]. The outcome is another project, the RAF9064, in partnership with IAEA and ESR entitled “Introducing the ESR- iGUIDE in Diagnostic Imaging Facilities in Africa”.

Much as all the above efforts have been fashioned to ensure justification of imaging procedures, studies indicate that there is a degree of inappropriate use of imaging ranging from 80 to 90% in some countries, while this could be higher others [37].

Action 2: Enhancing implementation of optimization of protection and safety through establishment of diagnostic reference levels (DRLs).

Africa has embarked on the process of establishing facility, and national DRLs partly through partnership with the IAEA but there are also individual, institutional and national initiatives.

Such a partnership has also resulted into sponsored workshops (Nairobi 2015 and Bulawayo 2019) in which personnel from Africa have been trained to establishing DRLs.

### Establishing facility and national DRLs

*Facility DRLs:* In South Africa, DRLs for Pediatric Computed Tomography [42] and DRLs for common CT examinations were established. In Nigeria, DRLs for abdominal CT examinations were established. DRLs in pediatric film-based radiography were established in Kenya [42–45].

*National DRLs: *Several African countries through IAEA regional projects RAF6053 and RAF9059 initiated processes of establishing national DRLs for high-dose imaging modalities. Algeria and Egypt have successfully established national DRL data for adult CT [46, 47]. Four countries (Ghana, Kenya, Namibia and Senegal) have jointly initiated a study to establish baseline for a regional DRL in adult CT. A National Diagnostic Reference Level Initiative for Computed Tomography Examinations in Kenya was undertaken [48] and there is an ongoing PhD project for Computed Tomography DRLs in Uganda aimed at National CT DRLs.

Action 3: Strengthen manufacturers’ role in contributing to the overall safety regime.

This has seen little progress. However, through the WHO, Africa is participating actively in formulation of medical equipment recommendations and specifications [49].

Action 4: Strengthen radiation protection education and training of health professionals.

The training sites and opportunities for radiation safety have increased with more formal and informal course in this area at all levels up to PhD as shown in Table 2. In addition, there is a drive towards non-ionizing imaging alternatives like ultrasound and MRI. Some institutions, with support from the World Federation of Ultrasound in Medicine and Biology (WFUMB) through its “Centers of Education” (WFUMB-CoES), have scale up training for radiation safety.

**Table 2:** Institutions for training of imaging personnel and medical physicists in Africa

Country	No. of institutions training radiologists	No. of institutions training radiographers	No. of institutions training sonographers	No. of institution’s training medical physicists
Kenya	3	1	1	0
Uganda	3	2	1	0
Tanzania	2	2	0	0
Nigeria	27 * (Fellowship training in 27 hospitals)	7	1	7
S. Africa	6	9	3	6
Ethiopia	4	4	0	1
Malawi	0	2	0	0
Angola				
Mozambique				
Cameroon	1	2	0	0
Algeria	8	7	0	7
Ghana	4 * (Fellowship training in 4 hospitals)	6	3	1
Cote d'ivoire	2	1	1	0
Swaziland	1	1	0	0
Zimbabwe	1	3	1	1

Effective cross-continental partnerships and affiliations have also been undertaken with the aim of standardizing practice for quality and sharing knowledge related to imaging and radiation safety. A case in point is the Ernest Cook Ultrasound Research and Education Institute (ECUREI) in Kampala, Uganda, trains ultrasound users in affiliation with Thomas Jefferson University-JUREI in the USA. There also other institutions in Africa that have been set up for training both fresh and in-service health professionals to ensure radiation safety but also to foster quality imaging services.

Table 2: imaging and medical physics training institutions in some countries Africa.

Despite the above efforts, the ratios of imaging human resource to population are still very low because few people undergo training and there is brain drain (Table 3).

**Table 3:** Numbers of imaging personnel for selected African countries

Country	Population	Radiologist	Radiologist to population	Radiographers	Sonographers	RT	Medical physicists	Year statistics obtained
Egypt	86,420,000	1250*	1:69,136					2012
Tunisia	10,850,000	450*	1:22,722					2012
Kenya	55,094,282	250	1:556,507	1700		42	7	2021
Uganda	42,460,000	55	1:772,000					2021
Tanzania	61,641,286	111	1:622,639	1200	10	–	5	2021
Nigeria	211,870,954	688	1:2,140,110	3221	399	19	100	2021
S. Africa	57,780,000	1200	1: 48,150	7910	545	700	166	2018
Ethiopia	118,158,035	300	1:1,193,515	2500		4	8	2021
D.R. Congo	84,070,000	42	1:2,001,666					2018
Malawi	19,681,633	3	1: 6,560,544	198	8		4	2021
Angola	30,810,000	30	1:1,026,999					2018
Mozambique	29,500,000	5	1: 5,900,000					2018
Sao Tome	211,028	2	1:105,514					2018
Guinea-Bissau	1,874,000	2	1: 937,000					2018
Cape-Verde	543,767	3	1:181,255					2018
Cameroon	27,287,100	176	1:275,627	380	*	4	2	2021
Algeria	44,738,446	1400	1:451,903	2800	US by radiologists	500	130	2021
Ghana	31,799,389	60	1:529,989	340	200	28	65	2021
Cote d'ivoire	27,090,000	327	1:273,636	300	US done by radiologists	3	2	2021
Swaziland	1,173,680	1	1: 1,173,680	37	11	0	0	2021
Zimbabwe	14,650,000	25	1: 586,000	386	37	10	5	2021

Table 3: Numbers of imaging personnel for selected African countries.

A presentation at the Global Summit on Radiological Quality and Safety (GSRQS) in 2015 showed that the proportion of radiologists who migrate outside their country to other countries is far higher for developing compared to developed countries and ranged from 2 to 35%. Immigration was mostly to developed countries although there is intra-continental migration [50]. It should also be noted that almost over 90% of radiologists in Africa are working in urban or periurban settings and only a minority work in rural areas. This means that oversight for quality and safety is less in the rural areas, where more than 80% of the population resides, yet there are radiology facilities in some of these areas. These rural facilities are largely manned by radiographers who take the images and report on them. Furthermore, equipment in these rural areas is often old, and not adequately maintained or serviced [51]. Such challenges affect education and training of health professionals in rural areas. The would-be solution would be to harness the possibility of application of ICT to enhance e-learning or training. However, this sector has also encountered a number of challenges, and thus, ICT application for learning is not possible [52].

### Training of medical physicists and their recognition as health professionals

Acute shortage of medical physicists prevails in Africa, with only 1040 medical physicists for the whole continent [53], albeit attempts to upscale training. Furthermore, their role is not well understood by their employers, governments and sector ministries. Medical physics engagement in mostly in radiotherapy, with very few in diagnostic imaging and nuclear medicine. Currently, only eleven countries (Algeria, Egypt, Ghana, Kenya, Libya, Morocco, Nigeria, South Africa, Sudan, Tunisia and Zimbabwe) have specialized postgraduate academic courses in medical physics and six of them (Egypt, Ghana, Morocco, Nigeria, South Africa, Zimbabwe) have clinical training programs. The International Centre for Theoretical Physics (ICTP) and University of Trieste in Italy, through their collaboration with the IAEA, also offer training to medical physicists from Africa and IAEA facilitated the establishment of the Federation of African Medical Physics Organizations (FAMPO) for supporting medical physics in Africa.

*Recognition of medical physicists as health professionals*: Only six (Algeria, Ghana, Namibia, South Africa, Tanzania and Zimbabwe) out of fifty-three countries in Africa claim to have legislative recognition of medical physicists as professionals. FAMPO, IAEA and WHO, however, continue to lobby for establishment of Medical Physics positions in Ministries of Health through regional workshops.

### Training in ultrasound

**Fig. 1:**
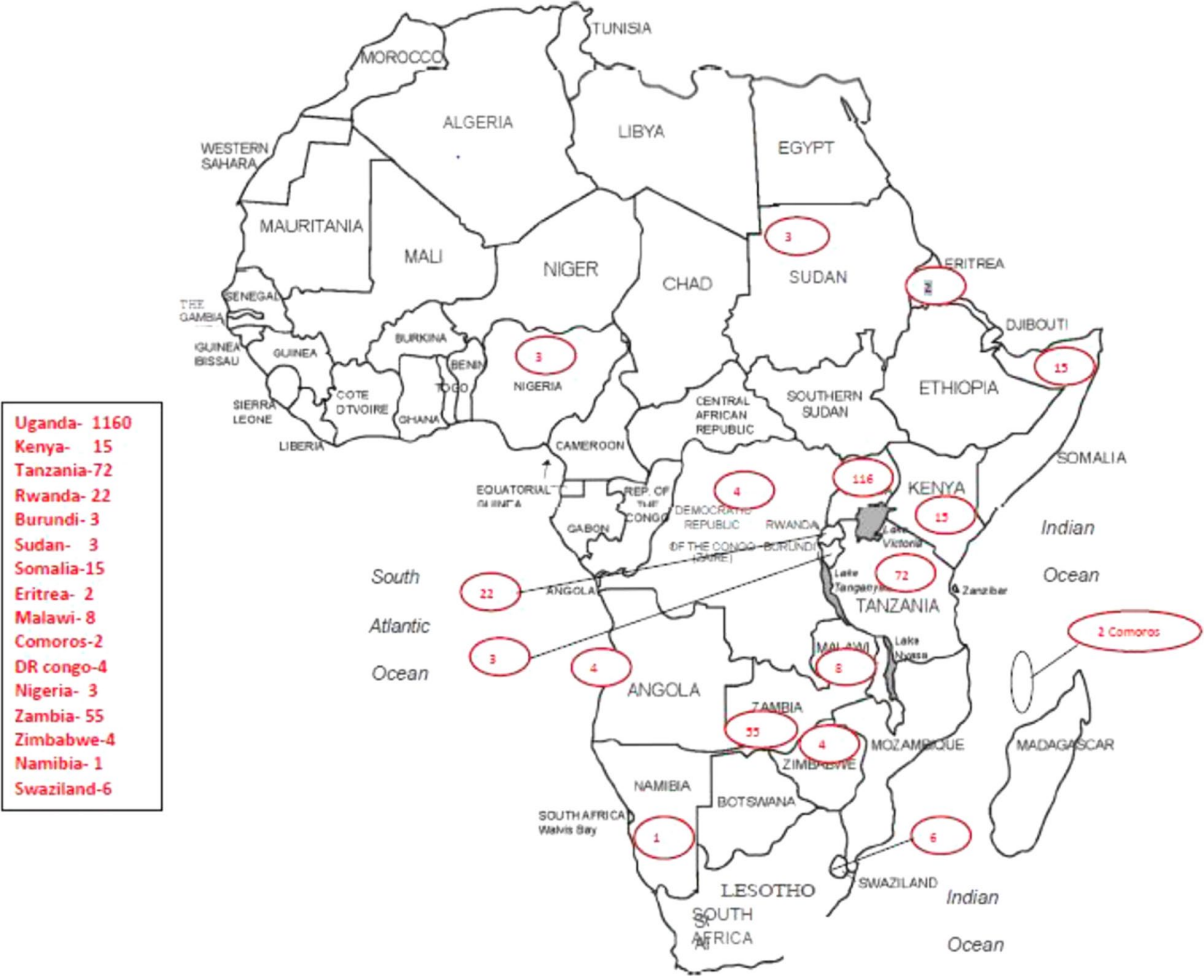
Countries in Africa where ECUREI trainees have come from and the number of trainees per country for the period 2012–2021

Ultrasound is a non-ionizing imaging alternative more widely available in African in comparison with CT and MRI, but the number of trained users still low mostly likely due to few training institutions. In sub-Saharan Africa, only South Africa and Uganda offer degree courses while Ghana offers short-term training in ultrasound [51, 54]. WFUMB set up Centers of Education (COEs) in Ethiopia, Kenya, Uganda, Nigeria, Togo and Northern Sudan aimed at short refresher courses. Such centers have played a great role in producing skilled personnel for rural and urban, national and foreign communities. An example is the Ernest Cook Ultrasound Research and Education Institute (ECUREI), located in Kampala, Uganda, a WFUMB-CoE, and also affiliate to Thomas Jefferson University–Philadelphia-USA which offers formal training courses leading to certificates, bachelors and masters’ degrees in ultrasound. ECUREI now records up to 1200 practitioners from 16 African countries trained to Master’s, Bachelor’s degree and Diploma levels in diagnostic ultrasound [55] as shown in Fig. 1.

Figure 1 shows countries in Africa where ECUREI trainees have come from and the number per country. ECUREI illustrates the training effectiveness, which can be achieved through locally initiated centers of excellence (CoE), in Africa, working in partnerships with international institutions and organizations [55]. Local conception and initiation are critical for sustenance of CoEs. The CoEs have been proposed as one way of improving access to imaging in LMICs [31].

Actions 6 Increase availability of global information on medical and occupational exposures in medicine.

The efforts to avail global information on medical and occupational exposures in medicine have been minimal, and this may be explained by low levels of awareness and knowledge for radiation safety and protection. The few exposure reports include: fluoroscopic radiation doses for Barium [56], radiation doses from c-arm fluoroscopy for surgeons in theatre [57], occupational doses for Ghanaian medical facilities [58] and adult patient doses in CT examinations in Algeria [47].

## African countries with nuclear regulatory bodies

To date, thirty-three countries in Africa have nuclear regulatory bodies and these constituted a forum of nuclear regulatory bodies [59]. Through these regulatory bodies, the national population dose trends can be monitored.

Actions 7, 8, 9 and 10; reporting and prevention of medical radiation incidents and adverse events, strengthening radiation safety culture in healthcare and Foster an improved radiation benefit-risk dialogue, strengthening the implementation of safety requirements (BSS) globally.

There has been little progress in these four actions basically because of inadequate awareness and prioritization. African countries in partnership with IAEA and WHO are promoting these actions through regional training workshops aimed at enhancing awareness, skills and setting of regional and national goals and strategies. Key resource persons (“champions”) are also identified and encouraged to pioneer interventions in their countries. The IAEA, IOMP, IRPA and WHO have produced a guidance document on radiation safety culture in health care. WHO has pioneered the skilling of health professionals in benefit–risk dialogue in pediatrics and published a tool “Communicating Radiation Risk in Pediatric Imaging” while undertaking user-training on the tool. There has been minimal action on the implementation the BSS apart from awareness creation through national and regional scientific meetings.

### Way forward to improving radiation safety in Africa

1. Evidenced-based Policy formulation, planning and financing, using evidence from local and global research and publications like the ISR endorsed publication (Frija et al., 2021) and the lancet commission.
2. Administration-system efficiency; efficient management of meagre imaging resources to minimize losses.
3. Audit and Accountability for services and resources; setting of local standards, proper systems, procedures and processes, plus regular clinical and financial audit for quality improvement, accountability and risk mitigation.
4. Adaptable systems and services to environmental fluctuations and disasters.
5. Awareness creation and continuing medical education through innovative exploitation of e-learning.
6. Availing appropriate and affordable and technologies with adoption of digital technologies including machine learning and artificial intelligence tools.
7. Amalgamating (integrating) imaging into the essential healthcare package and ensuring equitable access to quality and safe imaging.
8. Capitalizing on benefits of Private–Public-Partnerships. Significant proportion of imaging services and some training institutions in Africa are private, catering for the more affluent, but national governments, in partnership with these private facilities can deliver high-tech imaging and quality education to a wider populace with promotion of equity.
9. Collaboration with professional bodies, global agencies and organizations is a catalytic action which needs enhancement.
10. Education, training, research and innovation are catalytic actions which need enhancement.

## Conclusion

Quality and safety in imaging is important for Africa in sustaining and enhancing the gains Africa is experiencing with increased utilization of imaging. The drivers for quality and safety in Africa hinge uniquely on increasing accessibility. Workforce and infrastructure upscale to heighten accessibility require collaboration, training, education and innovation as catalysts.

Africa has made significant achievements within the lifetime of the Bonn Call for Action, which include raising radiation-safety awareness, rising interest and application of evidence-based radiation safety recommendations and guidance tools, establishing facility and national diagnostic reference levels (DRLs) and strengthening end-user education and training.

Limitations in workforce, awareness, prioritization of quality and safety and education opportunities remain major challenges. Solutions should target effectiveness, safety, patient-centeredness, timeliness, efficiency and equitability and integration of systems and services.

## Data Availability

Data sharing was not applicable to this article as no datasets were generated or analyzed during the current study.

## Abbreviations

BCA: Bonn Call for Action
BSS: Basic safety standards
CIG: Clinical imaging guideline
CoE: Centre of excellence
CT: Computed tomography
DRLS: Diagnostic reference levels
ESR: European Society of Radiology
GSRQS: Global Summit on Radiological Quality and Safety
IAEA: International Atomic Energy Agency
LMICs: Low- and middle-income countries
SDGS: Sustainable development goals
WFUMB: World Federation of Ultrasound in Medicine and Biology
